# Severe alcohol withdrawal during pregnancy or early postpartum: maternal and fetal outcomes

**DOI:** 10.1007/s00737-024-01531-4

**Published:** 2024-11-11

**Authors:** Shaun Daidone, Hayrunnisa Unlu, Asmaa Yehia, Nan Zhang, Osama A. Abulseoud

**Affiliations:** 1 Department of Psychiatry and Psychology, Mayo Clinic Arizona, 13400 E. Shea Blvd, Scottsdale, Phoenix, AZ 85259 USA; 2 Department of Medical Physiology, Faculty of Medicine, Mansoura University, Mansoura, Egypt; 3 Kern Center for the Science of Health Care Delivery, Mayo Clinic, Rochester, MN USA; 4 Department of Neuroscience, Graduate School of Biomedical Sciences, Mayo Clinic College of Medicine, Phoenix, AZ USA

**Keywords:** Alcohol withdrawal, Alcohol intoxication, Alcohol use disorder, Pregnancy, Pregnancy outcome

## Abstract

**Objective:**

Alcohol withdrawal syndrome (AWS) during pregnancy is under-researched despite growing concerns about increased alcohol use among pregnant women. This study aims to explore the severity of AWS and its impact on maternal and fetal outcomes.

**Methods:**

This retrospective study reviewed the medical records of patients admitted to the Mayo Clinic who underwent the CIWA-Ar protocol for AWS from June 2019 through June 2022. Pregnant women identified in this cohort had their pregnancy, labor, and neonatal data analyzed for alcohol-related complications and outcomes.

**Results:**

Out of the medical records reviewed, 8 cases involved pregnant women experiencing AWS. These cases showed a high severity of withdrawal symptoms, with a median peak CIWA-Ar score of 17 (IQR = 14). Maternal complications included a high rate of ICU admissions (37.5%; n = 3) and significant rates of miscarriage and stillbirth (37.5%; n = 3). Fetal outcomes were concerning, with 1 out of 5 (20%) neonates requiring NICU admission and experiencing conditions such as respiratory failure and neonatal abstinence syndrome. Developmental problems were noted in 2 out of 5 (40%) newborns.

**Conclusions:**

The findings highlight the severe implications of AWS during pregnancy, impacting both maternal and fetal health. The severity of AWS requires attentive clinical management and preventative interventions. Future research should focus on larger, prospective studies to better understand and address the risks associated with AWS in pregnant women and to improve health outcomes for mothers and their children.

**Article Highlights:**

• Severe AWS during pregnancy leads to high ICU admissions and adverse neonatal outcomes.

• 37.5% of pregnant women with AWS experienced miscarriage or stillbirth.

• 20% of newborns from mothers with AWS required NICU admission for serious conditions; 40% of newborns had developmental problems.

• Findings underscore the need for specialized treatment protocols to improve outcomes for pregnant women and their newborns.

## Introduction

Moderate alcohol drinking to control pain—specifically, the pain associated with ‘‘female complaints,’’ such as menstruation, pregnancy, childbirth, and menopause was recommended by 19th-century physicians (McClellan 2011). Two centuries later, Jones and Smith described fetal alcohol syndrome (Jones and Smith 1973) and raised public awareness of the deleterious long-term effects of drinking during pregnancy. Social alcohol drinking is common among women of childbearing age, leading to high rates of alcohol use during early pregnancy (Sundermann et al. 2021). Data from different national surveys show rates ranging between 7.6% and 17.7% (Centers for Disease, Control, and Prevention 2009; Agrawal et al. 2019; Havens et al. 2009; Tan et al. 2015; Crome and Kumar 2007; Centers for Disease, Control, and Prevention 2009) associated with 2.4% to 4.8% prevalence rates of fetal alcohol spectrum disorders (May et al. 2014). This bleak trajectory prompted the United States Surgeon General to issue a statement advising women who are pregnant or considering to get pregnant to abstain from using alcohol (Centers for Disease, Control, and Prevention 2009). Pregnant women who stop heavy drinking abruptly face the risk of alcohol withdrawal.

Alcohol withdrawal syndrome (AWS) is a common medical condition with varying presentations from mild hangover to life-threatening seizures, and delirium tremens (Amato et al. 2011; Campos et al. 2011; Muzyk et al. 2013; Ntais et al. 2005). The recent increase in hazardous drinking among women is accompanied by corresponding increase in alcohol-related emergency room visits and instances of AWS (Dawson et al. 2015; Grant et al. 2017; Keyes et al. 2008; Keyes et al. 2019; Schimmel et al. 2021; Seedat et al. 2009; White et al. 2015, 2018; Agabio et al. 2017; Kang et al. 2020). However, there is a significant lack of information about the prevalence, clinical manifestations, treatment response, and outcomes of AWS in women, including during pregnancy (Lee et al. 2019; Sanvisens et al. 2021; Unlu et al. 2023, 2024).

The harmful effects of alcohol consumption during pregnancy are well documented, encompassing a range of adverse maternal and fetal health outcomes. However, the impact of alcohol withdrawal during the prenatal and postpartum periods remains largely unexplored. This gap highlights the need for a better understanding of AWS's impact on maternal and fetal health (Amato et al. 2011; Campos et al. 2011; Kosten and O'Connor 2003; Muzyk et al. 2013; Ntais et al. 2005). Case reports on AWS during pregnancy are rare and mostly based on anecdotal evidence rather than thorough, systematic research. There is a critical need for more detailed analyses of alcohol consumption and withdrawal in pregnant women (McDonald et al. 2018; Bhat and Hadley 2015). The literature review reveals Bhat et al. and McDonald et al. to be some of the few comprehensive articles focusing on managing alcohol withdrawal in pregnant women (Bhat and Hadley 2015; McDonald et al. 2018). Bhat et al. offer preliminary recommendations and highlight the absence of defined safe alcohol levels during pregnancy, based on a case study and literature review (Bhat and Hadley 2015). McDonald et al. also emphasize the shortage of information on treating alcohol withdrawal in pregnancy through a clinical example and a systematic literature review (McDonald et al. 2018).

This small study aims to narrow the existing knowledge gap by providing an in-depth examination of eight cases involving alcohol consumption and withdrawal during pregnancy or the postpartum period. We hypothesize that alcohol intake during pregnancy correlates with adverse health outcomes for both the mother and child. Furthermore, we propose that pregnant women experiencing alcohol withdrawal are prone to more severe AWS manifestations, consistent with patterns observed among the general female population. This is based on the recognized increased susceptibility of women to the adverse effects of alcohol, even at lower levels of consumption and after shorter durations of alcohol use, compared to men (Hernandez-Avila et al. 2004; Keyes et al. 2010; Randall et al. 1999).

## Methods

The study was approved by the Institutional Review Board of Mayo Clinic (ID#22–008591). We collected the electronic medical records of all admissions at Mayo Clinic Health System during the interval from June 2019 through June 2022 for all patients who received an order for the implementation of the Clinical Institute Withdrawal Assessment for Alcohol–Revised (CIWA-Ar) protocol for alcohol withdrawal.

The CIWA-Ar protocol utilizes a CIWA-Ar score based on 10 items (nausea and vomiting; tremors; sweating; anxiety; agitation; tactile disturbances; auditory disturbances; visual disturbances; headache; and disorientation or clouding of the sensorium) to quantify the severity of withdrawal manifestations on a range of 0 to 67 points (Sullivan et al. 1989) and administer lorazepam based on the severity score. A score 0–9 do not receive lorazepam treatment; a score of 10–12 receives 1 mg of lorazepam orally or intravenously (PO/IV); a score of 13–14 receives 2 mg of lorazepam PO/IV; a score of 15–17 receives 3 mg of lorazepam PO/IV; and a score of 18 or greater receives 4 mg of lorazepam PO/IV. AWS symptoms are reassessed 30 min after lorazepam administration for further dosing. This approach has been associated with lowering benzodiazepine doses, which yields less sedation, shorter hospitalizations, fewer adverse reactions, and a decreased risk of respiratory depression in comparison to fixed-dose benzodiazepine treatment (Manasco et al. 2012).

All patient data were initially obtained from an electronic data extraction by senior data analysts. The active problem list at the time of admission was used to identify pregnant women during or shortly after the time of admission for alcohol withdrawal. Pregnancy, labor, and neonatal data were obtained by reviewing the medical records for each identified patient. Medical and psychiatric comorbidities and substance use profiles were all collected from the problem list and verified by reviewing the medical records. Hospital and Intensive Care Unit (ICU) length of stays (LOS) were calculated for each admission episode. The timestamps of individual CIWA-Ar assessments and benzodiazepine treatment were collected for each admission to identify the time to peak withdrawal severity and total benzodiazepine dose throughout the hospital LOS. Administered benzodiazepine doses were converted to lorazepam-equivalent doses (Brett and Murnion 2015). We used the revised Fenton growth chart for preterm infants to calculate the Z-Scores for head circumference and birth weight based on sex and gestational age (Fenton and Kim 2013).

The normality of continuous variables was assessed using the Shapiro–Wilk test. If normally distributed, data were presented as mean ± standard deviation (SD) or median, inter-quartile range (IQR), and range, while categorical data were expressed as percentages.

## Results

### Demographics

The study included eight patients with a mean age of 28.9(± 4.9) years. Six patients (75%) were single, 5 (62.5%) were unemployed, 5 (62.5%) were of non-Hispanic ethnicity and 4 (50%) were white. The patients' mean body mass index (BMI) was 29.3 (± 7.1) (Table 1).

**Table 1:** Demographics

	*n* (%) or mean ± SD	
Age [mean ± SD (Min–Max)]	28.9 ± 4.9 (20–32)	
Race	White	4 (50%)
	Others	3 (37.5%)
	Unknown	1 (12.5%)
Ethnicity	Hispanic	2 (25%)
	Non-hispanic	5 (62.5%)
	Unknown	1 (12.5%)
Marital status	Single	6 (75%)
	Married	1 (12.5%)
	Others	1 (12.5%)
Employment status	Unemployed	6 (75%)
	Employed	0 (0.0%)
	Unknown	2 (25%)
BMI [mean ± SD (Min–Max)]	29.3 ± 7.1 (20.1–37.4)	

### Medical and psychiatric comorbidities

Two patients (25%) had documented alcohol use disorder and 3 (37.5%) had other substance use disorder and 2 (25%) had tobacco use. Depression was reported in 6 (75%), generalized anxiety in 5 (62.5%) and history of childhood trauma or abuse in 4 (50%). Comorbid neurological conditions were reported in 4 (50%) patients and infection in 3 (37.5%) patients (Table 2).

**Table 2:** Medical and psychiatric comorbidities

	*n* (%)
Alcohol use disorder	2 (25%)
Other substance dependence	3 (37.5%)
Tobacco use	2 (25%)
Depression	6 (75%)
Generalized anxiety disorder	5 (62.5%)
Childhood trauma or abuse	4 (50%)
Neurological disorders	4 (50%)
Infections	3 (37.5%)
Endocrinological disorders	2 (25%)
Respiratory disorders	2 (25%)
Anemia	2 (25%)
Gastrointestinal disorders	2 (25%)
Sleep disorders	2 (25%)
Hypothyroidism	1 (12.5%)
Nutritional disorders	1 (12.5%)
History of altered mental status	1 (12.5%)
ADHD	1 (12.5%)
PTSD	1 (12.5%)

### Obstetrical history

Six (75%) patients had a history of abortion. Half of the patients (n = 4) had five or more pregnancies, three (37.5) had 3 full births, and two (25%) had four or more living children (Table 3).

**Table 3:** Obstetrical history

		*n* (%)
Number of pregnancies	Primigravida (1st pregnancy)	1 (12.5%)
	Multigravida (2–4 pregnancies)	3 (37.5%)
	Grand multigravida (≥ 5 pregnancies)	4 (50%)
Number of fullterm birth(s)	0	3 (37.5%)
	1	1 (12.5%)
	2	1 (12.5%)
	3	3 (37.5%)
History of preterm labor	0	6 (75%)
	1	2 (25%)
	2	0 (0%)
Number of previous abortion(s)	0	2 (25%)
	1	2 (25%)
	2	3 (37.5%)
	3	1 (12.5%)
Number of living children	0	3 (37.5%)
	1	1 (12.5%)
	2	1 (12.5%)
	3	1 (12.5%)
	≥ 4	2 (25%)

### Hospital course during AWS

The median (IQR) hospital stay for patients with alcohol withdrawal syndrome (AWS) was 45 (116) hours. ICU admission was required for 37.5% of the patients (n = 3), with a median ICU stay of 26 (34) hours. The median peak CIWA-Ar score was 17 (14), and the time from hospital admission to peak CIWA-Ar score was 10.5 (57) hours. Regarding medication, the median lorazepam dose equivalent administered within the first 24 h of hospitalization was 8 (15.8) mg, with the total dose for the hospital course being 19 (15) mg (Table 4).

**Table 4:** Hospital course during AWS

	*n* (%) or median (IQR)
Hospital Length of Stay [median (IQR) (hours)]	45 (116)
Required ICU admission	3 (37.5%)
ICU length of stay [median (IQR) (hours)]	26 (34)
Peak CIWA-Ar score [median (IQR)]	17 (14)
Time from hospital admission to peak CIWAAr score [median (IQR) (hours)]	10.5 (57)
Lorazepam dose equivalent within the first 24 h of hospitalization [median (IQR) (mg]	8 (15.8)
Lorazepam dose equivalent within whole course of hospitalization [median (IQR) (mg]	19 (15)
ALT (*n* = 4) [median (IQR) (U/L)]	39 (49)
AST (*n* = 4) [median (IQR) (U/L)]	37 (19)
Alkaline Phosphatase (n = 4) [median (IQR) (U/L)]	91.5 (55.5)

### Maternal and fetal outcomes

During pregnancy, one patient (12.5%) experienced diabetic ketoacidosis, 2 (25%) had pre-eclampsia, and another 2 (25%) had viral infections (HSV, COVID-19). Three patients (37.5%) lost conception: one miscarriage at 13 weeks and two stillbirths at 29 and 34.6 weeks. Five patients (62.5%) gave birth to living neonates. Four (80%) had normal vaginal delivery and one (20%) had a cesarean delivery. During labor, two patients (40%) were diagnosed with genital HSV, one (20%) had an active Group β Streptococcal infection, and another patient (20%) had severe preeclampsia. Neonatal percentiles show low birth weights, small head circumference, and shorter lengths.

Neonatal complications occurred in four (80%) cases. Specifically, one neonate required neonatal intensive care unit (NICU) admission, one (20%) had respiratory failure requiring intubation, another neonate had neonatal abstinence syndrome, and a fourth neonate had meconium aspiration with respiratory symptoms.

Developmental problems were reported in 60% of the cases (n = 3). Specific issues included speech delay (20%; n = 1), autism (20%; n = 1), feeding difficulties (20%; n = 1), seizure disorder (20%; n = 1), and trauma/fracture (20%; n = 1). Developmental delays were noted in 40% (n = 2), and laryngomalacia (20%; n = 1), torticollis (20%; n = 1), plagiocephaly (20%; n = 1), brachycephaly (20%; n = 1), hemiplegia (20%; n = 1), and congenital long QT syndrome (20%; n = 1) were also noted (Table 5).

**Table 5:** Maternal and fetal outcomes

		*n* (%)	
Maternal complications during pregnancy	Diabetic keto acidosis	1 (12.5%)	
	Pre-eclampsia	2 (25%)	
	Viral Infection (HSV, COVID-19)	2 (25%)	
Maternal complications during labor	Severe preeclampsia	1 (20%)	
	Group β Streptoccocal infection (active)	1 (20%)	
	Latent genital HSV (or history of)	2 (40%)	
Miscarriage or Stillbirth	Miscarriage at 13 weeks	1 (33.3%)	
	Stillbirth at 29 weeks	1 (33.3%)	
	Stillbirth at 34.6 weeks	1 (33.3%)	
Delivery method	Normal vaginal delivery	4 (80%)	
	Cesarean delivery	1 (20%)	
Gestational age at time of delivery (neonatal sex)	Neonatal percentiles		
	weight	length	head circumference
36w4d (F)	51	41	59
39w0d (M)	38	54	12
39w0d (F)	7	0	20
35w0d (M)	13	11	26
37w5d (M)	missing		
Neonatal complications	Any		4 (80%)
	Required admission to NICU		1 (20%)
	Meconium Aspiration With Respiratory Symptoms		1 (20%)
	Respiratory Failure/Intubation		1 (20%)
	neonatal abstinence syndrome		1 (20%)
Developmental problems	Any		3 (60%)
	Speech delay		1 (20%)
	Autism		1 (20%)
	Feeding difficulties		1 (20%)
	Seizure disorder		1 (20%)
	Trauma/fracture		1 (20%)
	Developmental delay		2 (40%)
	Laryngomalacia		1 (20%)
	Torticollis		1 (20%)
	Plagiocephaly		1 (20%)
	Brachycephaly		1 (20%)
	Hemiplegia		1 (20%)
	Congenital Long QT syndrome		1 (20%)

### Substance use histories

The ages of the patients at the time of delivery ranged from 20 to 32 years, with a mean age of 28.9 years. The interval between the alcohol withdrawal syndrome (AWS) encounter and the date of delivery varied widely, from a negative interval of -128 days (about 4.2 months after delivery) to a maximum interval of + 112 days (about 3.7 months before delivery). All patients (100%) had alcohol use during pregnancy, with the duration of alcohol use prior to pregnancy ranging from 0 days to 20 years.

Regarding other substance use during pregnancy, all 8 patients (100%) used substances in addition to alcohol. Nicotine use was the most common (75%), followed by amphetamines (50%), cannabis (25%), cocaine (25%), opioids (25%), and benzodiazepines (12.5%). Several patients also had a documented history of other substance use prior to pregnancy, with substances such as cocaine (37.5%), amphetamines (37.5%), and opioids (37.5%) reported. The number of prior hospitalizations related to substance use ranged from 0 to 5, with 5 of the patients (62.5%) having at least one hospitalization involving substance use prior to the episode of AWS (Table 6).

**Table 6:** Substance use histories

Patients age at date of delivery (years)	Interval between alcohol withdrawal encounter date and delivery date (days)	Alcohol use during pregnancy	Duration of documented alcohol use disorder prior to pregnancy (years, days)	Other substance use during pregnancy	Other substance use prior to pregnancy	Prior number of hospitalizations involving substance use
31	14	Yes	212 days	Nicotine	Nicotine	4
23	15	Yes	8 years, 178 days	Nicotine, Cannabis, Amphetsmines, Cocaine	Nicotine, Amphetsmines, Cannabis, Cocaine	1
20	0	Yes	None documented in chart	Cannabis, Cocaine	None	0
32	0	Yes	20 years, 237 days	Cannabis, Amphetsmines	Nicotine, Amphetsmines, Cannabis, Oploids	5
31	‒36	Yes	1 year, 119 days	Nicotine	Nicotine	0
30	‒128	Yes	9 years, 355 days	Nicotine, Amphetsmines	Nicotine, Amphetsmines	1
32	0	Yes	5 years, 25 days	Nicotine, Oploids	Nicotine, Oploids	0
32	112	Yes	19 years, 144 days	Nicotine, Amphetsmines, Benzodiazepines, Cocaine, Oploids	Nicotine, Amphetsmines, Benzodiazepines, Cocaine, Oploids	4

## Discussion

Our study presents one of the few detailed examinations of severe AWS during pregnancy and postpartum. Although there is limited existing data on this population, by comparing data from this research with our earlier published findings on adolescents and young adults, we can better understand AWS's impact across these demographics and during pregnancy, as both pregnant women and adolescents carry specific physiological and psychosocial challenges (Unlu et al. 2024). Adolescents, who are still undergoing significant development, often exhibit risk-taking behaviors and face high rates of suicidality and mental health disorders (Lees et al. 2020). Similarly, pregnant women undergo substantial physiological changes, such as increased alcohol clearance rate (Badger et al. 2005), which could theoretically intensify the impact of AWS and contribute to more severe withdrawal symptoms. The demographic differences between the adolescent cohort and the pregnant women in our study highlight the distinct nature of AWS in each group and comparing them underscores the need for tailored treatment strategies that account for their unique vulnerabilities. Additionally, the use of consistent metrics in both of our studies allows for a unique comparison of AWS severity and treatment responses across these groups.

In pregnant women, the median peak CIWA-Ar score was 17, far exceeding the adolescent group's median of 9, indicating more severe AWS in pregnant women (Unlu et al. 2024). The higher ICU admission rate in this study (37.5%; n = 3) compared to the adolescent group’s admission rate (22.3%; n = 33) mirrors this, and also suggests that AWS can escalate to life-threatening conditions more rapidly in pregnancy than in the general population (Bhat and Hadley 2015; McDonald et al. 2018). Additionally, pregnant women required a higher initial benzodiazepine dose (8 mg of lorazepam equivalent) than adolescents (2 mg), which also corresponded to the greater severity of their symptoms. However, they had a shorter median hospital stay (45 h) compared to adolescents (68.8 h), which was possibly due to more aggressive early treatment (Unlu et al. 2024).

The severity of AWS in pregnant women may be due to physiological changes during pregnancy. Animal studies provide insights into a possible mechanism by showing that chronic alcohol exposure in pregnant rats leads to higher ethanol levels in both maternal and fetal compartments, confirming ethanol’s ability to cross the placental barrier (Través et al. 1995; Través and López-Tejero 1994; Beattie 1986). Additionally, these studies observed a reduction in liver alcohol dehydrogenase activity, essential for ethanol metabolism, which suggests an impaired detoxification process during pregnancy (Través et al. 1995; Través and López-Tejero 1994). These findings propose a biological basis for the more severe AWS symptoms in pregnant women compared to other groups (Través et al. 1995; Través and López-Tejero 1994).

The majority of the patients had significant comorbid psychiatric conditions, with depression (75%; n = 6) and generalized anxiety disorder (62.5%; n = 5) being particularly prevalent. This aligns with existing literature that suggests a high co-occurrence of mood disorders and substance use disorders (Grant et al. 2017; Yonkers et al. 2017). Medical comorbidities were also commonly observed, including infectious diseases (37.5%; n = 3) and neurological disorders (50%; n = 4). Psychiatric comorbidities are known to play a significant role in the outcome of AWS. For example, comorbid depression is associated with increased alcohol craving severity and post-withdrawal relapse (Andersohn and Kiefer 2004). While there is limited data on the specific impact of psychiatric disorders on AWS during pregnancy, it is well-established that these disorders can exacerbate withdrawal symptoms, likely due to heightened physiological stress and dysregulation of the autonomic nervous system (Adinoff et al. 1998). Anxiety and mood disorders during pregnancy are also linked to adverse outcomes such as preterm labor, preeclampsia, and poor neonatal health, compounding the risks already associated with AWS (Abulseoud et al. 2023). Additionally, these psychiatric conditions often lead to increased substance use, contributing to more severe AWS and worse maternal and fetal outcomes (Caleyachetty et al. 2014; Volkow et al. 2019). The high rates of NICU admissions and developmental problems seen in our study may be influenced by this interaction between psychiatric disorders, substance use, and the demands of pregnancy.

Substance use during pregnancy, beyond alcohol consumption, presented an additional layer of complexity. All eight patients reported using substances other than alcohol during pregnancy, with nicotine, amphetamines, and cannabis being the most prevalent. This finding aligns with existing literature, which highlights the high co-occurrence of multiple substance use in pregnant women with alcohol use disorder (Caleyachetty et al. 2014; Volkow et al. 2019). Polysubstance use during pregnancy exacerbates maternal and fetal risks, increasing the likelihood of neonatal complications such as neonatal abstinence syndrome and respiratory distress (Shankaran et al. 2007; Bailey and Diaz-Barbosa 2018). These additional substances may have contributed to the heightened severity of AWS observed in our cohort, as well as adverse maternal and neonatal outcomes.

Alcohol withdrawal carries well-known complications due to increased CNS activity, including autonomic instability, cognitive distortions, anxiety, and seizures (Bhat and Hadley 2015; McDonald et al. 2018; Kosten and O'Connor 2003). These complications are thought to be caused by the abrupt cessation of GABA inhibition and a significant increase in glutamate levels (Thomas and Riley 1998). These biochemical changes during AWS, particularly during peak withdrawal stages, exacerbate the risk of severe complications from alcohol use alone, including congenital defects like fetal alcohol syndrome and miscarriages (Lemoine et al. 2003; Polygenis et al. 1998; Thomas and Riley 1998). In pregnant patients, these biological alterations can also include preterm labor, fetal distress, and placental abruption (Bhat and Hadley 2015; McDonald et al. 2018). Indeed, in our study, pregnancy complications were prevalent, including miscarriages, stillbirths, severe preeclampsia, and Group β streptococcal infections, which were significantly more prevalent than in the general population (Bhat and Hadley 2015; Bhuvaneswar et al. 2007; Lemoine et al. 2003). This highlights the high-risk nature of pregnancies complicated by AWS, and while there is extensive literature on congenital defects like fetal alcohol syndrome, limited data exists on how the severe stress of AWS may heighten these risks by further altering maternal physiology and thereby increasing the teratogenic effects on fetal development (Polygenis et al. 1998; Testa et al. 2003).

Neonatal outcomes were also concerning, with 1 in 5 (20%) newborns requiring NICU admission and others suffering from conditions such as respiratory failure and neonatal abstinence syndrome. Developmental delays and other long-term conditions were also observed, supporting the evidence that the impacts of maternal AWS extend beyond the immediate postpartum period, potentially affecting long-term child development (Polygenis et al. 1998; Testa et al. 2003; Trofimov et al. 1996). One possible mechanism by which alcohol withdrawal could harm the developing fetus is related to the NMDA receptor, which binds the neurotransmitter glutamate. This receptor is vital for normal brain development (Thomas and Riley 1998). During withdrawal, overstimulation of the NMDA receptor may cause the death of brain cells (Thomas and Riley 1998) and subsequent behavioral changes related to in-utero exposure to alcohol (Thomas and Riley 1998). Another consideration is that while benzodiazepine use is essential for managing severe withdrawal symptoms, it also increases the risk of miscarriage, congenital malformations, and neonatal complications. Specifically, studies have shown that prenatal exposure to benzodiazepines may increase the risk of cleft palate, as well as neonatal complications like respiratory distress and neonatal benzodiazepine withdrawal after birth (Bellantuono et al. 2013; Freeman et al. 2018; Grigoriadis et al. 2019, 2020; Meng et al. 2024; Sheehy et al. 2019; Wikner et al. 2007; Yonkers et al. 2017). These withdrawal symptoms may require prolonged NICU stays and specialized care, adding to the already significant burden of managing maternal AWS during pregnancy. Additionally, prolonged use of benzodiazepines during pregnancy has been associated with later developmental delays and cognitive impairments in children (Grigoriadis et al. 2020). Given these risks, there is a growing need to explore alternative treatments for managing AWS during pregnancy, such as non-benzodiazepine medications like gabapentin or the use of adjunctive therapies like vitamin and supplementation to mitigate harm to the fetus (Leggio et al. 2008).

The results of this study should be viewed in light of its strengths and limitations. The study provides a detailed description of the withdrawal phenotype, medical and psychiatric comorbidities, and maternal and fetal outcomes in eight patients. While this number is still very small, it is the largest sample size in the literature to our knowledge. The retrospective nature of the study limits the ability to obtain a more detailed alcohol or other substance use history. Another limitation of this study is the absence of a formal control group, such as pregnant women without AWS or non-pregnant women experiencing AWS. A comparative analysis could provide valuable insights into the differential impacts of AWS in these groups. However, due to the retrospective nature of this study and the small sample size, this was not feasible. Future research should aim to include a control group to allow for a more comprehensive understanding of how pregnancy specifically affects the severity and outcomes of AWS. In addition to the lack of a control group, several other limitations must be considered when interpreting the findings of this study. The retrospective design also introduces potential biases, such as selection bias, where only severe cases of AWS may have been captured, and information bias due to reliance on medical records, which may lack complete details. Variations in the management of AWS, including different benzodiazepine dosing and timing, could also influence the outcomes. Additionally, inconsistent follow-up durations for neonatal outcomes may have affected the detection of long-term developmental issues. Polysubstance use and psychiatric comorbidities further confound the findings, as mentioned previously, and these factors may have independently contributed to adverse maternal and fetal outcomes. Future prospective studies with standardized treatment protocols and comparison groups are needed to better understand these risks.

Despite these limitations, our report lends more evidence to the well-known fact that AWS during pregnancy has severe implications, leading to significant maternal and neonatal complications. At-risk pregnant women require diligent monitoring, preventive strategies, and specialized care to mitigate these risks. Several key recommendations can be made to improve maternal and neonatal outcomes based on the findings of this study. First, early and routine screening for alcohol use in pregnancy is essential to identify at-risk women before the onset of severe AWS. Once identified, timely and effective treatment must be prioritized to manage AWS and mitigate associated risks. Special attention should be given to integrating addiction services into prenatal care, ensuring that pregnant women receive comprehensive, multidisciplinary support throughout their pregnancy. This should include addiction counseling, mental health services, and medical management of withdrawal symptoms. Additionally, ongoing monitoring of both maternal health and fetal development is crucial to detect complications early, allowing for interventions that can prevent more severe outcomes for both mother and child. Still, larger studies using national data are needed to identify risk factors, societal prevention measures, and pregnancy-safe pharmacological treatment options to improve mothers' and infants' outcomes.
